# Forensic Psychiatry in Iran

**Published:** 2013

**Authors:** Seyed Mehdi Saberi, Gholam Reza Mirsepassi

**Affiliations:** 1 Psychiatrist, Assistant professor, Legal Medicine Research Center.; 2 Psychiatrist, President of Iranian Psychiatric Association, Psychiatry and Behavioral Sciences Research Center, Mazandaran University of Medical Sciences, Sari, Iran.

**Keywords:** Criminal Responsibility, Legal Medicine, Iran Jurisdiction System

## Abstract

In Iran, department of forensic psychiatry is one of the special units of Legal Medicine Organization concerned with individuals who demonstrate psychological and psychiatric problems. The duties of forensic psychiatrists in the department are, performing psychiatric examinations and determining mental competence of two major groups of referrals: Individuals who are involved in a legal problem related to civil law and individuals who are involved in criminal responsibility and/or forbearance of punishment such as offenders and prisoners.

One of the worries of the Iran jurisdiction system is the absence of a secure mental hospital devoted to the irresponsible mentally ill criminals. In fact, there is no forensic inpatient unit available in the country.

## Introduction

In Iran, there are three distinct powers: legislative, executive and judiciary. The judiciary is an independent power, the protector of the rights of the individual and society and responsible for the implementation of justice. The head of Iran judiciary power is appointed by the supreme leader for a period of five years. He, in turn, appoints the head of the Supreme Court and the Attorney General. The judiciary power, in addition to many other duties, has the responsibilities for Investigation, prosecution and punishment of criminals in accordance with the Islamic penal code (1).

The Legal Medicine Organization of Iran, as an independent body of the Judiciary power, was established in 1993. It provides expert opinions in the field of forensic medicine on the basis of scientific, legal and jurisprudential principles to relevant authorities using its qualified experts and modern technologies in order to help in detection of truth and establishing justice in the society (2).

In the past, there were some famous specialists at the head of legal medicine office (the previous name of legal medicine organization) of Iran (3). Professor Abdolhossein Mirsepassi, was the only psychiatrist who assumed the responsibility of directing legal medicine office for three years (1948-1950) (4). The late Professor Gholam Reza Bahrami, an eminent psychiatrist and a member of Tehran medical faculty, had an interest in forensic psychiatry and used to teach this subject at Faculty of law, Tehran University. Also, Professor Shokrollah Tarighati was one of the pioneers of forensic psychiatry in Iran. His book named “criminal psychiatry” in Farsi, was the first book on the subject and used by many students of law and postgraduate medical doctors in psychiatry.

From 1980 to 1992, some psychiatrists of psychiatric department of medical schools were assigned to the duties of forensic psychiatrists such as psychiatric evaluation of mentally ill patients for determining competence, criminal responsibility and punishment forbearance. From 1992, psychiatrists who had interests in forensic psychiatry have been hired in legal medicine center of Tehran after theoretical and experimental education in forensic sciences. Thereafter, gradually some specific courses were established in legal medicine organization of Iran.

Forensic psychiatry department is one of the special units of the organization concerned with individuals who demonstrate psychological and psychiatric problems. At present there are 10 forensic psychiatry departments in the country, where the largest one is located in Tehran (5). Since Tehran department is not only the largest of all, but is the referral center for all of the departments in other provinces, we are going to discuss its structure in details:

In forensic psychiatry department of Tehran, there are five full time psychiatrists, one clinical psychologist with M.A. degree and four with B.A. degrees. The department is headed by Seyed Mehdi Saberi ( one of this paper’s authors). The duties of forensic psychiatrists in the department are, performing psychiatric examinations and determining mental competence of two major groups of referrals:

Individuals who are involved in a legal problem related to civil law such as guardianship, financial exploitation, and doubtful mental ability for decision making about marriage or divorce or child custody, legal abortion, job exhaustion and seeking legal permission for sex reassignment surgery because of suffering from gender identity disorder.

Individuals who are involved in criminal responsibility and/or forbearance of punishment such as offenders and prisoners.

The main duty of forensic psychiatrists is determining criminal responsibility of persons with mental disorder who committed a crime.

It is worth mentioning that according to Islamic penal code, article 51, “if the offense is committed during insanity, whatever the degree of the insanity; the offender will be exempted from criminal responsibility” (6).

Also, according to Article 52, “if, during committing a crime or after committing the crime, the offender is insane and the insanity and its dangerous condition is diagnosed by a specialist, by the order of prosecutor[the offender] will be detained in an appropriate place until the insanity is cured…”

The forensic psychiatrist tasks are not limited to the performing of the mentioned duties. Giving expert opinion in the court for the necessity of involuntary admission and treatment, remaining a criminal patient in the hospital for long duration and evaluating the risk of violence are some examples of such duties performing by forensic psychiatrist in the department. The department, also, deals with the complaints made by patients against psychiatrists and other members of the psychiatric team.

It should be emphasized that one of the worries of the Iran jurisdiction system is the absence of a secure mental hospital devoted to the irresponsible mentally ill criminals. In fact, there is no forensic inpatient unit available in the country. The forensic patients are detained in prison (and/or general psychiatric hospital) and the psychiatrists visit them there. In addition to beds in mental health facilities, there are 45,400 beds in other residential facilities such as homes for persons with mental retardation, detoxification inpatient facilities; homes for the destitute, etc(7). It is clear that necessity of existence a secure mental hospital is unavoidable. Recently, some efforts have been executed by a group of Iranian psychiatrists to write a compiled mental health act (8). It could be the first step to establish a secure mental hospital and improve the situation of forensic psychiatry in Iran.
